# Feasibility study to evaluate cycloidal vibration therapy for the symptomatic treatment of intermittent claudication

**DOI:** 10.1186/s40814-019-0514-6

**Published:** 2019-11-17

**Authors:** Leanne Atkin, John Stephenson, Karen Ousey

**Affiliations:** 1 School of Human and Health Sciences, University of Huddersfield, Huddersfield, UK; 2 Mid Yorkshire NHS Trust, Wakefield, UK; 3 Institute for Skin Integrity and Infection Prevention, School of Human and Health Sciences, University of Huddersfield, Huddersfield, UK

**Keywords:** Peripheral arterial disease, Intermittent claudication, Vascular disease, Cycloidal vibration therapy, Vibropulse

## Abstract

**Introduction:**

Intermittent claudication (IC) is the most common symptom of peripheral arterial disease. Previous research has suggested that cycloidal vibration therapy (CVT) may induce angiogenesis and improvements in circulation. The objective of this feasibility study was to explore trial design and acceptability of the protocol to provide data to estimate the parameters required to design a definitive randomised control trial. This feasibility study specifically aimed to assess recruitment rate; attendance rates at baseline and follow-up; and safety, tolerability, and compliance with therapy device and additionally, to consider the potential efficacy of CVT as a novel treatment for intermittent claudication.

**Methods:**

Patients with intermittent claudication (IC) were recruited and CVT was applied at home for 30 min twice a day for a period of 12 weeks. Primary outcomes were pain-free walking time (PFWT) and maximum walking time (MWT) after 12 weeks of treatment. Secondary outcomes included the ankle-brachial index and ankle systolic blood pressure. Participants were assessed during active therapy phase at baseline, week 4, week 8, and week 12.

**Results:**

Thirty-four participants with IC were recruited: 30 (88%) male and 4 (12%) female. The rate of recruitment was 2.4 participants per month from a standard-size district general hospital. No participants left the study during the activity therapy stage, and no participant failed to attend their follow-up appointment. The general compliance with CVT was high. No participants dropped out during the treatment phase. The mean age of all participants was 68 years (IQR 60–75 years). Substantive improvements were seen in a comparison of differences in times to PFWT and MWT, in ABPI, and in systolic leg pressure in the treated leg. There was no evidence of a substantive difference from baseline in systolic leg pressure in the untreated leg. There were no immediate or delated treatment safety concerns of documented adverse effects with the treatment, all patients completed the required 12-week course indicated a high degree of patient acceptability.

**Conclusion:**

The statistically significant and substantive improvements from baseline after 12 weeks observed in PFWT and MWT in participants experiencing IC are comparable to improvements seen from other treatment options such as supervised exercise as reported by Stewart et al. (N Engl J Med 347:1941–1951, 2002). The substantive improvement in systolic leg pressure in the treated leg and the concurrent absence of a substantive change in systolic leg pressure in the untreated leg over the same period suggests a causative effect.

This study has provided novel information relating to the number of potential eligible participants for a further research trial and potential association between CVT and improved symptoms. Additionally, it has established that CVT treatment is highly acceptable, as indicated by no participant drop-out in the treatment phase, and may potentially offer an alternative treatment option for patients experiencing IC. Furthermore, this study has assessed the variability of the primary outcome measure which provides vital information needed to calculate sample sizes for any future studies.

In conclusion, this study has established the feasibility of using CVT to improve patients’ symptoms of IC and provides essential information which will contribute to the design of future research investigating whether the improvements seen are directly related to CVT.

## Introduction: background

Peripheral arterial disease (PAD) is caused by the development of atherosclerosis in the lower limb arteries and is associated with increased morbidity and mortality. PAD is underdiagnosed, undertreated, and poorly understood by the medical profession [1, 2]. A common symptom of PAD is intermittent claudication (IC), a severe cramp-like pain in the muscles of the lower legs experienced when walking. This is caused by a reduction in blood supply, leading to lack of oxygenation of the muscle cells. These symptoms severely limit exercise performance and walking ability/distance and as such negatively affect patients’ quality of life [3]. PAD affects approximately 20% of the population over 55 years of age in the Western world, with an estimated 27 million sufferers in North America and Europe [4].

The National Institute for Health and Care Excellence (NICE) [5] and the Scottish Intercollegiate Guidelines Network (SIGN) [6] have published guidelines for the management of PAD. The guidance states that all patients with IC should be offered a supervised exercise programme as a first line of intervention and that further treatment options, such as angioplasty or medication, should only be offered when a supervised exercise programme has failed to lead to satisfactory improvements in symptoms. Supervised exercise has been shown to improve peripheral circulation that can provide symptomatic relief and improve walking distance before pain is experienced [7]. However, currently, supervised exercise programmes are not widely available in the National Health Service (NHS) across the United Kingdom (UK) [8]. This is reported to be due to the running costs, lack of resource, and poor patient compliance with exercise programmes [8, 9].

Due to the limitations of the treatment options currently available, alternative therapies to improve patients’ symptoms of IC have been explored. A potential alternative to current treatments is cycloid vibration therapy (CVT), a low-frequency and -amplitude form of oscillatory non-invasive energy.

The transmission of CVT into the tissues generates a range of mechanical forces and stresses on the vascular endothelial cells, which has been shown to induce the release of NO. Vascular-produced NO is an important vasodilator which regulates vascular smooth muscle tone and maintains healthy blood flow. Additionally, the presence of NO is the mediator for angiogenesis (the formation of new blood supply) [10]. CVT has been shown to increase NO levels, leading to increased blood flow [11, 12].

Vibropulse (Vibrant Medical) is a portable machine which delivers CVT. Vibropulse is promoted as a therapy for cellulitis, venous leg ulcers, and lower limb oedema [13–15]. The device is a rectangular soft pillow-style pad, approximately the size of the lower leg, which is connected to a transformer powered via mains electricity.

This research focuses on whether the stimulation of these mechanisms through CVT in the lower limb at the point of, and surrounding area of, arterial disease could improve blood flow; therefore, increasing arterial perfusion and thus increasing patients’ walking distance. There have been limited case studies [16, 17] demonstrating these improvements, and the majority of these case studies have been performed on patients with critical limb ischaemia. If CVT is effective in improving patient symptoms, this would support the use of CVT as an alternative treatment for patients with IC, especially those who are not able to access or undertake a supervised exercise programme and/or those not wishing to be exposed to the risks or side effects that angioplasty or medication bring.

### Introduction: specific objectives

The objective of this feasibility study was to explore trial design and acceptability of the protocol to provide data to estimate the parameters required to design a definitive randomised control trial. The primary objectives of the trial were as follows:

1. Feasibility objectives
  - Recruitment. To assess how many patients accepted the invitation to participate in the research
  - Follow-up. To assess attendance of participants to baseline visits and subsequence follow-up visits
  - Compliance. To investigate safety, tolerability, and compliance with therapy device
2. Clinical objectives: To consider the potential efficacy of CVT as a treatment of IC
  - Primary outcomes. PFWT and MWT after 12 weeks of treatment
  - Secondary outcomes. Ankle-brachial index and ankle systolic blood pressure

## Methods

### Trial design

The study design was a prospective, single-patient group feasibility study to investigate the impact of cycloidal vibration therapy in patients with IC and to estimate the change in participants’ pain-free walking time (PFWT), maximum walking time (MWT), ankle-brachial pressure index (ABPI), and systolic leg pressure.

### Participants

Thirty-four participants were recruited from a single tertiary vascular unit within Mid Yorkshire NHS Trust, United Kingdom, over a period of 14 months between July 2014 and September 2015. Inclusion criteria were (1) age over 18 years, (2) experiencing lower limb claudication caused by PAD (as defined per NICE guidelines [5]), (3) categorised to Fontaine’s classification stage II a or stage II b, (4) palpable femoral pulses and triphasic Doppler signals within femoral artery on affected slide, and (5) ability to provide informed written consent. Exclusion criteria included (1) prescription of medication for the treatment of intermittent claudication, e.g. cilostazol or naftidrofuryl, (2) pregnancy, (3) deep vein thrombosis within the last 6 months, (4) active lower limb soft tissue or bone infection, (5) patients with active cancer, and (6) unstable lower limb of joint structures.

Ethical approval was granted by the local Research Ethics Committee (REC reference 17/YH/0080) and the trial was registered on NIHR Clinical Research Portfolio (CARD 3410, registered 23 May 2014: https://www.nihr.ac.uk/research-and-impact/nihr-clinical-research-network-portfolio/). The research was conducted in accordance with the ethical standards of the Committee on Human Experimentation from the Declaration of Helsinki 1975.

Participants were recruited from a single vascular out-patient clinic, but only when the researcher was available/present. A convenience sample was obtained by clinical staff who scanned and identified potential participants meeting the study inclusion/exclusion criteria. Those meeting the criteria were provided printed information sheets about the purpose of the study. Participants were then approached at the end of their clinical consultation to assess if they were willing to be involved. Those wanting to participate were then provided further verbal information by the researcher and written consent was obtained. There was no blinding of either research staff or the participants, as within the feasibility protocol all participants received active therapy.

### Intervention

**Fig. 1:**
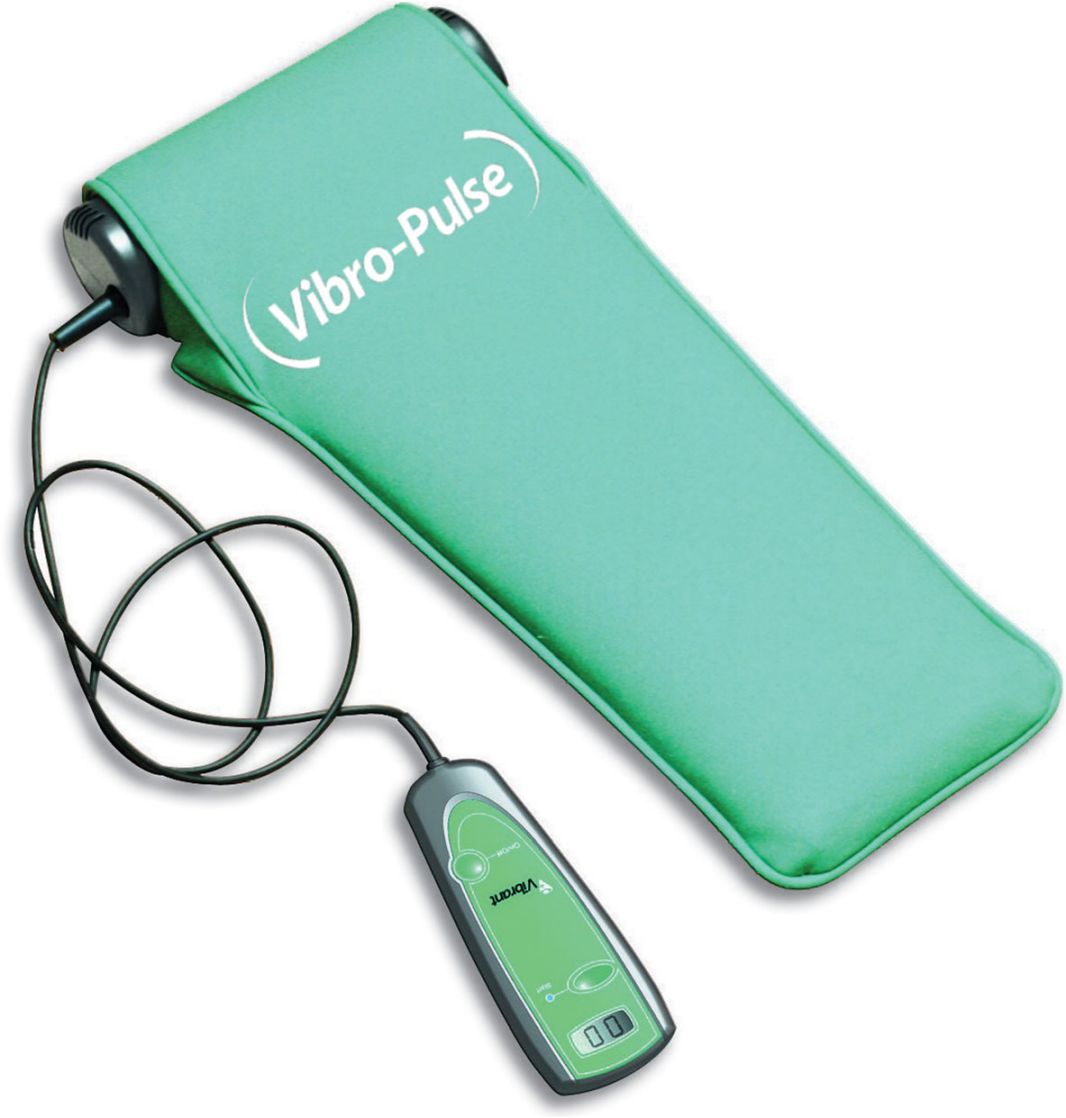
Vibropulse machine

Participants were supplied with a Vibropulse machine (Fig. 1) to be used in their own homes and asked to apply CVT for the recommended time of 30 min twice a day for a period of 12 weeks to the lower limb at the point of suspected arterial narrowing or occlusion, as assessed clinically/through imaging. A study period of 12 weeks was selected as this is the same length of time patients are asked to attend supervised exercise programmes. Thirty minutes’ vibration is recommended for the treatment of conditions such as venous ulceration, oedema management, and the treatment of cellulitis [18]. There was previous published evidence of physiological changes within the skin after 5 min of vibration with increased nitric oxide level [12] and with increased blood flow being evident after 15 min of vibration [11]. Previous studies exploring the use of Vibropulse in the treatment of cellulitis, oedema, or ulceration have reported positive results using the product twice or three times a day [13–15].

### Feasibility outcomes methods and analytical methods

#### Recruitment

The recruitment rate consisting of the eligibility and consent rates were recorded descriptively.

#### Follow-up

Participants were required to attend follow-up appointments within a hospital out-patient environment at week 4, week 8, and week 12. Numbers of those who failed to attend appointments and/or were lost to follow-up were recorded.

#### Compliance

Tolerability and compliance with the therapy device were assessed in terms of usage and repeat usage of the intervention by the participant, as indicated by the device counter on the machine. Any issues with safety were captured by means of event/serious event reporting in line with Good Clinical Practice standards. Individual participant use of the CVT machine was recorded within the machine device counter, allowing usage to be monitored. If participants fully adhered to the recommended twice a day usage for a period of 12 weeks, the device counter should read 168. A degree of variation was allowed in the form of a 20% leeway either side of the 100% compliant value of 168. This degree of variation was based on methodology for medication compliance [19]. It is acknowledged that compliance in relation to medication is different to compliance with treatments such as CVT, but in the absence of data relating to the degree of appropriate variation of use in relation to non-medication treatments, the 20% leeway of compliance was deemed appropriate.

### Clinical outcomes

The choice of study measures was guided by previous research and the recommendations within the Transatlantic Society Consensus guidelines on the management of PAD [3] and the National Institute Clinical Excellence guidelines relating to PAD [5]. Outcomes selected were exploratory outcomes exploring the potential efficacy of CVT as a treatment for IC. The sample was summarised descriptively at baseline, reporting age, gender, smoking status, medications, blood pressure, past history of PAD, and previous PAD interventions (surgical, endovascular, or conservative).

#### Primary outcomes: PFWT and MWT after 12 weeks of treatment

Individuals with IC have limited exercise and walking capacity, and as such, the severity of disease and changes in condition are measured via walking ability [5]. The primary outcome measures of the study were changed from baseline in PFWT and MWT at 12 weeks (the end of the treatment phase). A simple walking test was performed to document any real-life changes in the patients’ ability to walk. Participants were instructed to walk at their normal speed, to report when they started to feel pain, and to continue walking until the pain becomes unbearable and forced them to rest. The researcher walked with them around the circuit. The circuit was entirely indoors and flat with no inclines or stairs. The route varied at each assessment, so participants did not have any prior knowledge of the distance they last walked. Time recording was started on the participant’s first step. PFWT was recorded as the time at which the participant first expressed pain and MWT was recorded as the time the participant was forced to stop walking. Both the PFWT and MWT test were stopped at 8 min (480 s). If a participant was able to walk further than this, the maximum time in seconds was recorded as a censored observation.

Time-to-event (survival) analysis was conducted on the outcomes of PFWT and MWT at baseline and at the end of active therapy. This technique was adopted to reflect expected data censoring: a certain proportion of patients were expected to record censored times by reaching the temporal limit of the test without experiencing pain. As the size of the sample precluded the concurrent assessment of the effects of multiple factors on walking times in a semi- or fully parametric context, the non-parametric Kaplan-Meier method was used to assess survival. Due to anticipated skewness of data, measures were reported in terms of median, rather than mean, values from baseline.

#### Secondary outcomes: ankle-brachial index and ankle systolic blood pressure

Ankle-brachial pressure index (ABPI) and systolic leg pressure were recorded at baseline and after 12 weeks. Where participants’ leg pressures were incompressible, a pressure of 280 mmHg was recorded, as this is the maximum on the sphygmomanometer gauge. All assessments were undertaken before any treatment at baseline and at 4, 8, and 12 weeks.

### Sample size

Since this was a feasibility study, sample size calculations were not undertaken. However, it was estimated that a sample size of between 30 and 40 participants would provide a large enough sample to inform of the practicalities of delivering the research protocol. The final number recruited was determined by a pragmatic approach of setting a specific time period of 14 months for recruitment of participants.

## Results

### Feasibility outcome: recruitment

During the 14 months’ recruitment period of the trial, 121 patients were screened. Of these, 87 were excluded because they either declined participation (*n* = 22) or they did not meet the inclusion/exclusion criteria (*n* = 65). Thirty-four participants were enrolled to the study, and all received 12 weeks of CVT. On average, the rate of recruitment was 2.4 participants per month from a standard-size district general hospital.

### Feasibility outcome: follow-up

No participants left the study during the activity therapy stage, and no participant failed to attend their follow-up appointment. However, 4 participants failed to complete every walking assessment, due to a variety of reasons, including chest pain on exercise, fear of falling, and muscular-skeletal/joint pain.

### Feasibility outcome: compliance

Patients’ compliance to any treatment is important. Within this study, the participants were provided with the device to use at home, and the general compliance with CVT was high. No participants dropped out during the treatment phase. This indicates the high degree of participant acceptability of the treatment, which is in stark contrast to supervised exercise programmes, where attrition loss during the treatment phase is very common [20]. The high compliance to CVT is a great advantage to ensure resources are used appropriately and to maximise treatment benefits.

Twenty-six participants (76%) were compliant with the CVT treatment. Eight participants (24%) had usage outside this level, but interestingly half of these participants had a higher level of usage than that recommended. It is possible that these participants were using the machine more frequently than was recommended.

### Baseline characteristics of the study population

All participants were white Caucasian. The age of participants ranged from 51 to 83 years, with a mean age of 68 years (median 68.5 years) and an interquartile range (IQR) of 60–75 years. Participant demographics and past medical history are summarised in Table 1.

**Table 1:** Participants’ demographics and co-morbidities

Variable	Frequency (valid %)
Gender	
Male	30 (88.2%)
Female	4 (11.8%)
Diabetes	
Yes	9 (26.5%)
No	25 (73.5%)
Hypertension	
Yes	23 (67.6%)
No	11 (32.4%)
History of CVA/TIA	
Yes	1 (2.9%)
No	33 (97.1%)
History of IHD/Angina/MI	
Yes	12 (35.3%)
No	22 (64.7%)
Smoking status	
Current	6 (17.6%)
Previous	23 (67.6%)
Never	5 (14.7%)

### Clinical outcomes: primary outcomes—PFWT and MWT after 12 weeks of treatment

**Fig. 2:**
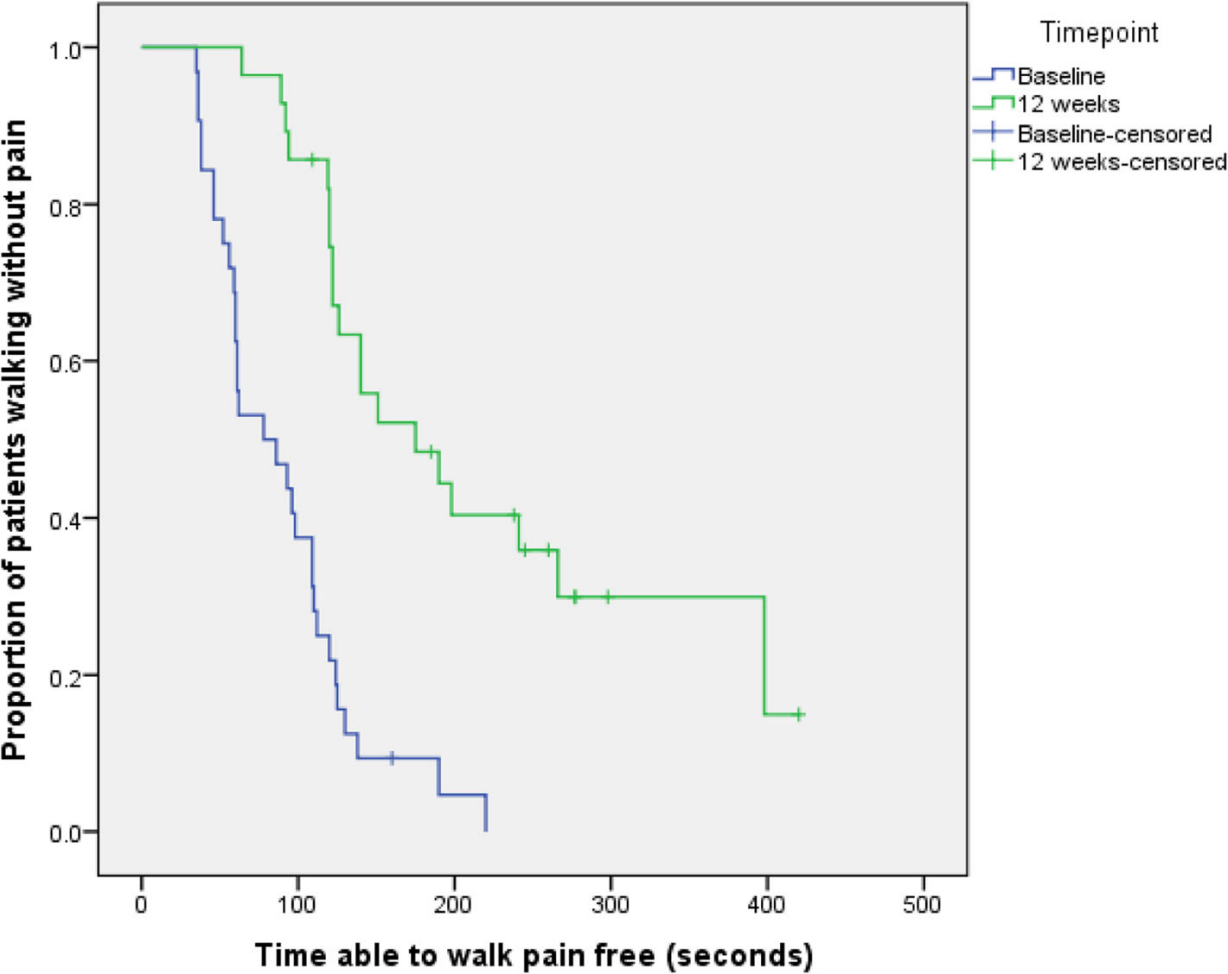
Time-to-event analysis of PFWT baseline and PFWT at week 12

The median pain-free walking time at baseline, calculated from all 34 patients, was 82 s (range of 35 to 220 s; IQR 53 to 118 s). Thirty participants (88%) provided valid measurement of PFWT at week 12. Mean change in PFWT from baseline to 12 weeks was 97.7 s (SD 85.0 s) (Fig. 2, Table 2).

**Table 2:** Summary statistics of PFWT as measured at different time points

	Number	Minimum	Maximum	Mean	Std. deviation
Baseline PFWT (s)	31	35	220	88	46.7
Week 4 PFWT (s)	29	60	300	136	60.7
Week 8 PFWT (s)	30	72	360	161	68.0
Week 12 PFWT (s)	28	64	420	186	90.4

**Fig. 3:**
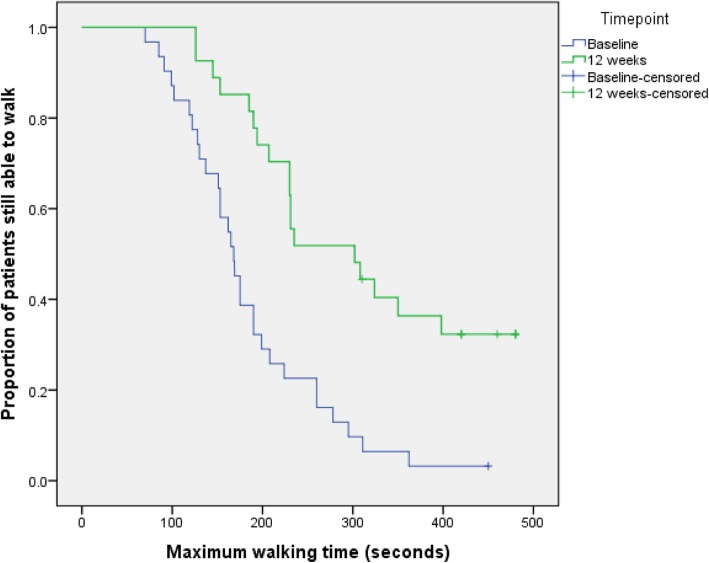
Dot plot of PFWT as measured at various time points

The dot plot (Fig. 3) illustrates the change in PFWT over time in mean pain-free walking times (with associated 95% confidence intervals) showing monotonically increasing trend in pain-free walking time within the active therapy period from baseline to 12 weeks.

**Fig. 4:**
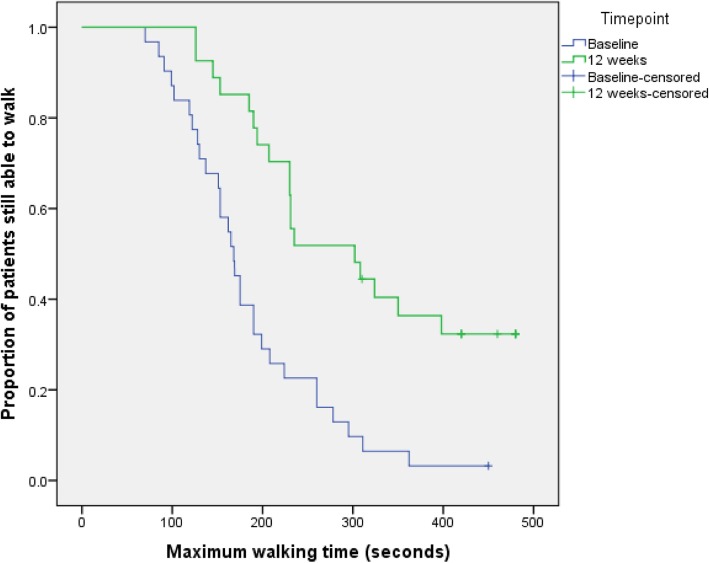
Time-to-event analysis of MWT baseline and MWT at week 12

The median maximum walking time at baseline, calculated from all 34 patients, was 186 s (range 70 to 450 s; IQR 128 to 224 s). Twenty-seven participants (79%) provided a valid measurement of MWT at week 12. Mean change in MWT from baseline to 12 weeks was 98.9 s (SD 108.1 s) (Fig. 4, Table 3).

**Table 3:** Summary statistics of MWT as measured at different time points

	Number	Minimum	Maximum	Mean	Std. deviation
Baseline MWT (s)	30	70	450	186	87.0
Week 4 MWT (s)	28	83	480	224	105.0
Week 8 MWT (s)	29	102	480	266	108.6
Week 12 MWT (s)	26	126	480	294	118.8

**Fig. 5:**
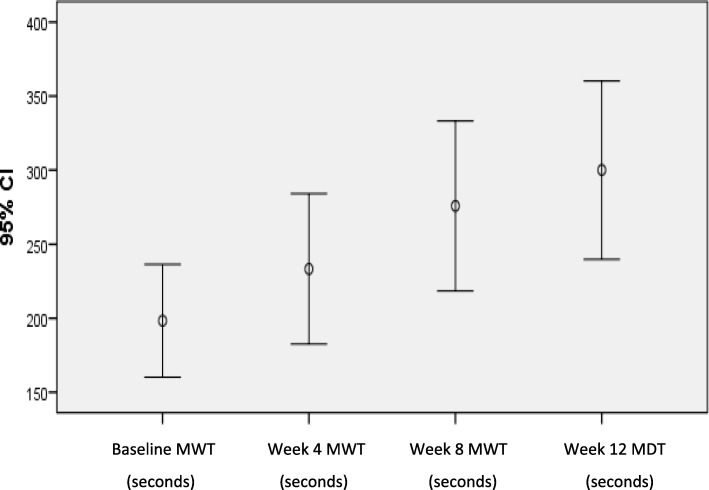
Dot plot of MWT measured at multiple time points

Mean maximum walking times (and associated 95% confidence intervals are illustrated in a dot plot (Fig. 5), illustrating the monotonically increasing trend in maximum free walking time with number of weeks from baseline.

### Clinical outcomes: secondary outcomes ankle-brachial index and ankle systolic blood pressure

Thirty participants provided valid ABPI measurements to compare ABPI at baseline and at the end of the treatment phase. Mean ABPI in the treated leg at baseline was 0.64 (SD 0.18); mean ABPI in the treated leg at week 12 was 0.71 (SD 0.21), hence a substantive difference of 0.071 (SD 0.187).

Twenty-four (71%) of the participants had an increase in systolic leg pressure during the treatment phase. Pressure remained static for 2 participants (5%) and deteriorated for 8 participants (24%). The average increase was 12%, ranging from − 40 to 90%.

Thirty-two participants provided valid measurements of systolic leg pressure at baseline and week 12. Highest systolic pressure in the treated leg at baseline was 111 mmHg (SD 47.7); highest systolic pressure in the treated leg at week 12 was 120 mmHg (SD 52.1), hence a difference of 8.53 (SD 21.2).

Highest systolic pressure in the untreated leg at baseline was 137 mmHg (SD 52.9); highest systolic pressure in the untreated leg at week 12 was 139 mmHg (SD 50.1), hence a difference of 2.38 (SD 22.5).

Hence, results show improvements in systolic leg pressure of the treated leg in the 12 weeks from baseline, and no change over the same time period in the untreated leg; suggesting that the changes to systolic leg pressure are a direct result of CVT.

## Discussion

The objective of this feasibility study was to explore trial design and acceptability of the protocol to provide data to estimate the parameters required to design a definitive randomised control trial. The research explored the relationship between CVT and PAD and established the feasibility of using CVT to improve patients’ symptoms of IC. The results of this study highlight that following 12 weeks of active treatment there were improvements demonstrated in participants’ PFWT. The degree of improvement in PFWT was substantive, even though the study was of a feasibility design and hence not powered accordingly to detect significant effects. On average, participants’ PFWT increased by 163% from baseline, and this level of improvement is comparable to improvements seen from other treatment options such as supervised exercise [21]. Stewart et al. [21] reported an average improvement of 120% from supervised exercise. Furthermore, a systematic review of the evidence for the Cochrane group by Lane et al. [22] showed supervised exercise has a positive effect on walking ability in the range of 50 up to 200%. The level of improvements found within this study is at the higher end of this scale.

Improvements were also seen in participants’ MWT. There was on average an 83% improvement in MWT. This level of increase remains within the scale of improvements seen with exercise programmes [22].

The low baseline PFWT (88 s) and MWT (186 s) highlights the severe impact IC can have on activity levels. However, the overall mean changes in PFWT (to 186 s at week 12) and MWT (to 294 s at week 12) could be considered to be clinically meaningful, particularly for MWT which was very close to 300 s. This level of exercise tolerance has been shown to be relevant to the individual and a predictor of the ability to progress towards health prompting walking and normative exercise values [23].

Further significant effects were observed during the analysis of certain secondary outcomes, again suggesting a substantive effect of the therapy. Assessment of change in participants’ lower limb perfusion showed evidence of a substantive difference between ABPI at baseline and at the end of week 12. Furthermore, substantive changes were seen in the treated leg when comparing systolic leg pressure at baseline and week 12. However, in the untreated leg, there was no evidence of a substantive difference. This physiological change established that improvements seen in walking distance are more likely to be due to improvement in blood supply rather than the result of a placebo effect.

In this study, a number of participants failed to complete the walking tests. Difficulties were encountered in completion of the walking test due to significant co-morbidity from coexisting cardiovascular disease, the elderly population, and issues with balance/increased risk of falling. This reinforced the difficulties with this group of patients being able to participate in exercise therapy. For future studies, it would be worthwhile amending the inclusion/exclusion criteria so that potential participants are required to undertake a form of cardiovascular screening/walking assessment to ensure that all potential candidates are able to fully participate in the research. However, this process of screening has limitations, as this will result in a study group which is not truly representative of the whole claudication group and it may exclude patients with the most severe limitations on walking distance and those with multiple co-morbidities. Nevertheless, acknowledging the limitations of this approach by defining precise populations (that may not fully reflect the whole IC group) will provide detailed information on outcomes and any results could be extrapolated to the wider population.

No participants dropped out during the treatment phase. This indicates the high degree of participant acceptability of the treatment, which is in stark contrast to supervised exercise programmes, where attrition loss during the treatment phase is very common [20]. The high compliance to CVT is a great advantage to ensure resources are used appropriately and to maximise treatment benefits.

Although a sample size calculation might be expected to be an output from a pilot study, the significant results obtained indicate that the sample of the pilot is more than adequate to detect effects between baseline and 12 weeks. In fact, even at 4 weeks, the effect is sufficiently large to be detected with the pilot sample size.

### Limitations

The study has several possible limitations. A major limitation of this study was in relation to being a single-arm trial and absence of a control group; therefore, any improvements reported cannot be attributed directly to the intervention. Further, limitations are the choice of a simple walking test to measure walking time both PFWT and MWT. This method of testing has limitations due to issues with reliability, repeatability, comparability with other studies, and repeatability.

However, whether CVT is responsible for these improvements cannot be proven or disproven in this feasibility study. Nevertheless, it has been established that there may be an association between the improvements and CVT. To increase confidence in the hypothesis that CVT improves PFWT and MWT in patients with IC requires further research in the form of a randomised control trial. Another issue arising from the lack of a comparator group is that the significance levels reported from the log-rank tests conducted to compare MWT and PFWT at baseline and 12 weeks are likely to be under-reported, due to the clustering inherent in the within-participant comparisons. However, this should have no substantive effect on conclusions, as statistical significance was already obtained.

It would have also strengthened the study if statistical modelling were utilised to investigate whether there was any link between participants change in PFWT/MWT and peripheral perfusion.

The potential for observer bias is also acknowledged, as the researcher was not blinded and had prior knowledge of the research aims, disease status, and intervention. As such, these can all influence data recording [24]. The researcher tried to minimise the risk of bias by following standardised protocol for enrolment and follow-up. The potential of reporting bias and observer bias could be reduced by implementing blinding to future studies.

A further limitation is due to the study being conducted at a single NHS site with a single researcher who designed, delivered, collected data, and analysed the results. This was inevitable since the research was conducted by a single researcher as part of the PhD process. This does reduce the generalisability of the findings. However, as this was a feasibility study, the research was not intended to evaluate outcomes nor infer generalisability.

## Conclusion

PAD is a common chronic condition and affects patients’ quality of life, and the treatment goal is to relieve pain and improve patient quality of life. Existing treatments to reduce symptoms of IC include medication, exercise, angioplasty, or bypass surgery [25]. Exercise therapy can be in the form of simple advice asking the patient to regularly walk through the pain. However,this form of unsupervised exercise fails to address the barriers to walking faced by patients with IC [26]. Supervised exercise has been shown to offer improvements in patients’ symptoms of IC and help with some of the barriers to exercise such as fear and motivation [27]. However, even though supervised exercise is an effective treatment, it is often underused due to lack of availability and many patients being unwilling or unsuitable to participate. This study has established that CVT is a potentially viable alternative treatment to supervised exercise which eliminates many of the factors which hinder supervised exercise from being used.

There is emerging evidence of the effects of CVT on the improvement of nitric oxide production, improved blood flow and increased rate of angiogenesis [11, 12, 28]. This increased blood perfusion would reduce symptoms of IC. This is the first study investigating the feasibility of using CVT as a treatment for IC and has provided novel information relating to length/positioning of treatment, potential association between CVT and improved symptoms, and described research methodology required for future research. In conclusion, this study has established the feasibility of using CVT to improve patients’ symptoms of IC.

## Recommendations for future research

This research has highlighted a number of issues which warrant future research. Further research should examine the effectiveness of CVT, ideally in a multi-centre double-blind randomised controlled trial design, potentially using a placebo dummy machine, using a greater number of researchers to recruit and collect the data. This should include a health economic evaluation which can be compared to current treatment options. This would provide valuable information about the translation and transition of CVT into everyday healthcare.

## Data Availability

Source data is secured and stored at the NHS site.
